# Caskin2 is a novel talin- and Abi1-binding protein that promotes cell motility

**DOI:** 10.1242/jcs.262116

**Published:** 2024-05-16

**Authors:** Wei Wang, Paul Atherton, Maaike Kreft, Lisa te Molder, Sabine van der Poel, Liesbeth Hoekman, Patrick Celie, Robbie P. Joosten, Reinhard Fässler, Anastassis Perrakis, Arnoud Sonnenberg

**Affiliations:** ^1^ Division of Cell Biology, The Netherlands Cancer Institute, Plesmanlaan 121, Amsterdam 1066 CX, The Netherlands; ^2^ Department of Molecular and Clinical Cancer Medicine, Institute of Systems, Molecular and Integrative Biology, The University of Liverpool, Liverpool L69 7BE, UK; ^3^ Proteomics Facility, The Netherlands Cancer Institute, Amsterdam 1066 CX, The Netherlands; ^4^ Division of Biochemistry, The Netherlands Cancer Institute, Amsterdam 1066 CX, The Netherlands; ^5^ Department of Molecular Medicine, Max Planck Institute of Biochemistry, 82152 Martinsried, Germany; ^6^ Oncode Institute and Division of Biochemistry, The Netherlands Cancer Institute, Amsterdam 1066 CX, The Netherlands

**Keywords:** Caskin2, Talin, Focal adhesion, Tension, KANK, Cortical microtubule stabilizing complex, Abi, WAVE

## Abstract

Talin (herein referring collectively to talin 1 and 2) couples the actomyosin cytoskeleton to integrins and transmits tension to the extracellular matrix. Talin also interacts with numerous additional proteins capable of modulating the actin-integrin linkage and thus downstream mechanosignaling cascades. Here, we demonstrate that the scaffold protein Caskin2 interacts directly with the R8 domain of talin through its C-terminal LD motif. Caskin2 also associates with the WAVE regulatory complex to promote cell migration in an Abi1-dependent manner. Furthermore, we demonstrate that the Caskin2–Abi1 interaction is regulated by growth factor-induced phosphorylation of Caskin2 on serine 878. In MCF7 and UACC893 cells, which contain an amplification of *CASKIN2*, Caskin2 localizes in plasma membrane-associated plaques and around focal adhesions in cortical microtubule stabilization complexes. Taken together, our results identify Caskin2 as a novel talin-binding protein that might not only connect integrin-mediated adhesion to actin polymerization but could also play a role in crosstalk between integrins and microtubules.

## Teaser

**Summary:** Caskin2 interacts with talin via a C-terminal LD motif, and with the SH3 domain of Abi1 via its proline-rich region. The Caskin2–Abi1 interaction functions to regulate cell motility.

## INTRODUCTION

Cell migration is essential for several biological processes including embryogenesis, wound repair and tissue homeostasis, and also underpins metastasis of cancer cells. Migrating cells form actin-driven protrusions that are linked to the underlying extracellular matrix (ECM) by integrin-mediated adhesions. Activated integrins, which engage with ECM components, dynamically cluster at the leading edge. The assembly and disassembly of integrin-mediated adhesions is regulated by actin polymerization; furthermore, many proteins capable of influencing actin polymerization are known to bind to integrin-associated proteins. Thus, integrins and polymerizing actin are linked by complex feedback systems that remain poorly defined.

Actin polymerization at the leading edge is driven by the Arp2/3 complex, which is regulated by a complex of five proteins [WASF1 or WASF2, CYFIP1 or CYFIP2, ABI1 or ABI2, NCKAP1 and HSPC300 (also known as BRK1)] called the WAVE regulatory complex (WRC) (Rottner et al., 2021). Meanwhile, integrins are indirectly connected to actin via multidomain proteins including talin (herein referring collectively to talin 1 and 2) and vinculin, which undergo force-mediated conformational changes that affect their dynamics and the recruitment of additional proteins (Jansen et al., 2017). Talin cycles between the cytosol, where it remains auto-inhibited, and the plasma membrane, where it binds integrin tails upon activation (Goult et al., 2013a). The N-terminal FERM domain of talin binds integrins, phosphatidylinositol 4,5-bisphosphate (PIP_2_), Arp2/3 and F-actin (at actin-binding site 1; ABS1), whereas the long C-terminal rod domain consisting of 13 α-helical bundles (R1–R13) (Calderwood et al., 2013) contains two additional actin binding sites (ABS2 and ABS3; Atherton et al., 2015) and 11 vinculin-binding sites (Calderwood et al., 2013) that are exposed upon force-mediated unfolding of the talin rod domains (Goult et al., 2018; Yao et al., 2016). In addition to integrin, actin and vinculin, multiple other proteins have been identified to interact with talin, including paxillin and DLC1 (also known as ARHGAP7) (Zacharchenko et al., 2016), α-synemin (Sun et al., 2008), tensin3 (Atherton et al., 2022), KANK-family members (Bouchet et al., 2016; Sun et al., 2016), RIAM (Lee et al., 2009) and CDK1 (Gough et al., 2021). The interaction of KANK family proteins with talin has recently attracted considerable attention (Bouchet et al., 2016; Guo et al., 2023; Li et al., 2023; Rafiq et al., 2019; Sun et al., 2016) as the KANK proteins can connect to microtubules through a complex of proteins, referred to as the cortical microtubule stabilization complex (CMSC; Noordstra and Akhmanova, 2017), which localizes at the plasma membrane. These CMSCs are strongly clustered around focal adhesions (FAs) at the leading edge and might play an important role in their turnover (Rafiq et al., 2019; Sun et al., 2016).

Here, we identified Caskin2 as a novel interactor of both talin and Abi1. The talin interaction is mediated by a C-terminal LD motif, whereas Abi1 binding occurs via the Abi1 SH3 domain interacting with the proline-rich region of Caskin2. Furthermore, we show that the Caskin2–Abi1 interaction is regulated by phosphorylation of S878 of Caskin2, which occurs downstream of EGFR and MEK1 and MEK2 (MEK1/2, also known as MAP2K1 and MAP2K2, respectively) activation. Meanwhile, overexpression of Caskin2 promotes cell motility. Finally, we show that in Caskin2 is a component of the CMSCs. Overall, our results identify Caskin2 as a scaffold protein connecting integrin-mediated adhesions to actin polymerization and microtubules.

## RESULTS

### Caskin2 is a scaffold protein in proximity to cell–ECM adhesion sites

**Fig. 1.**
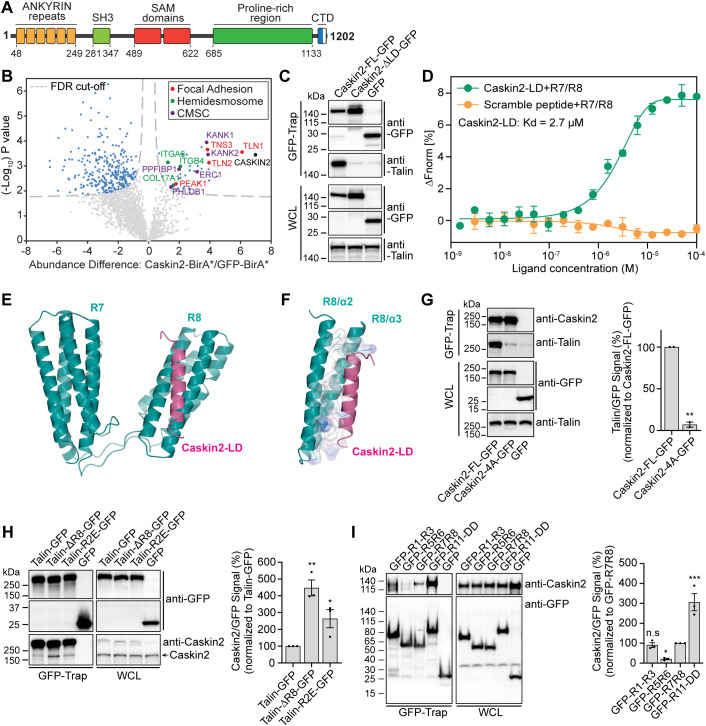
**Caskin2 binds directly to talin via a C-terminal LD motif.** (A) Schematic of caskin2; the C-terminal domain (CTD) contains an LD motif (white bar). (B) Proximity biotinylation assays were performed with PA-JEB/β4 keratinocytes ectopically expressing either Caskin2 fused to the biotin ligase BirA* or GFP–BirA*, as a negative control. The volcano plot shows the results from three independent experiments [threshold false discovery rate (FDR): 0.01 and S0: 0.1]. Significant proximity interactors of Caskin2 and GFP are indicated in light blue (GFP interactors, left and Caskin2 interactors, right), red (FA proteins), green (hemidesmosome proteins) or purple (CMSC components). (C) GFP-trap pulldown of Caskin2–GFP and Caskin2-ΔLD–GFP expressed in GE11^tetON β1^ cells, followed by western blotting for talin and Caskin2. (D) MST assay demonstrating binding of Caskin2-LD peptide to the talin R7/R8 peptide (*n*≥5). Peptide with scrambled amino acid sequences was used as negative control. Error bars show mean±s.d. (E) X-ray structure of the talin-R7R8 (residues 1358–1653, cyan) fragment in complex with Caskin2-LD peptide (residues 1187–1202, magenta). (F) Structure analysis show the hydrophobic interaction (with surface charge distribution) of Caskin2-LD (magenta ribbon) with talin-R8 (cyan ribbons). Residues from both talin R8 and Caskin2-LD peptide that form the interface between the two molecules are shown in sticks. The electrostatic surface of these residues are shown [blue for positive, red for negative (not present) and white for neutral]. (G) GFP-Trap pulldown of GFP, Caskin2–GFP and Caskin2-4A–GFP expressed in COS-7 cells. Proteins in the GFP-trap sample and whole-cell lysate (WCL; input) were detected by western blotting using antibodies against talin, Caskin2 and GFP. Graph shows quantification of talin binding to Caskin2-FL–GFP or Caskin2-4A–GFP from GFP-Trap pulldown experiments; *n*=3 independent repeats. **P<0.01 (two-tailed unpaired *t*-test). (H,I) GFP-Trap pulldown in COS-7 cells expressing Caskin2 (without a GFP tag) together with talin–GFP, talin-ΔR8–GFP and talin-R1523E/K1530E–GFP (H) or the indicated GFP-tagged talin polypeptides (I). Proteins in the GFP-trap sample and WCL (input) were detected by western blotting using antibodies against Caskin2 and GFP. *P<0.05, **P<0.01, ***P<0.001 (one-way ANOVA with uncorrected Fisher's LSD multiple comparisons test). Error bars in G–I are mean±s.e.m. Western blots in C, G, H and I are representative of three independent experiments.

We previously identified Caskin2 (*CASKIN2*) in the proximity of integrin α6β4 in PA-JEB/β4 keratinocytes (β4-deficient keratinocytes reconstituted with wild-type β4) by a proximity-dependent biotin identification (BioID) assay combined with mass spectrometry-based proteomics (te Molder et al., 2020). Caskin2 is a relatively unexplored scaffold protein composed of six ankyrin repeats (ANK), an atypical (Kwan and Donaldson, 2016) Src homology 3 (SH3) domain and tandem sterile α-motifs (SAM) domains, followed by an extended disordered proline-rich region (PRR) and a conserved C-terminal domain (CTD) (Marchler-Bauer et al., 2015; Smirnova et al., 2016) (Fig. 1A). Caskin2 is widely expressed in adult tissues, whereas expression of Caskin1 is restricted to the brain (Human Protein Atlas; https://www.proteinatlas.org/ENSG00000167971-CASKIN1). Whereas the majority of studies on Caskin2 have focused on its role in neuronal cells, its interaction partners, localization and biological function in non-neuronal cells remain unexplored.

To gain a better understanding of the function and localization of Caskin2, we first used BioID in combination with mass spectrometry to identify Caskin2-interacting proteins. For these experiments, we used PA-JEB/β4 keratinocytes given that it was in these cells that we originally identified Caskin2 as a proximal protein of α6β4. After stable expression of Caskin2 fused to the promiscuous biotin ligase BirA* (Caskin2–BirA) in PA-JEB/β4 keratinocytes, biotinylated proteins were captured with streptavidin beads and subjected to mass spectrometry. A total of 36 Caskin2-interacting and proximal proteins were identified, including several components of FAs [talin 1, 2 (Klapholz and Brown, 2017), tensin 3 (Atherton et al., 2022) and PEAK1 (Zuidema et al., 2022a)], hemidesmosomes (ITGA6, ITGB4 and COL17A1; te Molder et al., 2021) and cortical microtubule stabilizing complexes [KANK1 and KANK2, PPFIBP1 (liprin-β1), PHLDB1 (LL5α) and ERC1 (also known as ELKS) (Noordstra and Akhmanova, 2017)] (Fig. 1B; Table S1). Taken together, these data suggest that Caskin2 is in close proximity to different matrix adhesion sites and associated complexes.

We confirmed the subcellular localization of Caskin2 by expressing Caskin2 tagged at the C-terminus with GFP, in PA-JEB/β4 keratinocytes. Confocal microscopy revealed that Caskin2–GFP partially colocalized with both integrin β4 and talin (Fig. S1A), a FA protein that is also a proximity interactor of integrin α6β4 in keratinocytes (te Molder et al., 2020).

### Caskin2 binds directly to talin via a C-terminal LD-motif

Several proteins interact with talin via leucine-aspartic acid motifs (LD motifs) – short helical motifs that facilitate protein–protein interactions with the helical bundles of the talin rod domains – with the talin R7R8 domains being the best of the characterized talin LD-binding domains (Zacharchenko et al., 2016) (Fig. S1B). Both Caskin1 and Caskin2 contain an LD motif in their conserved C-terminus (Fig. S1C), suggesting they might be binding partners of talin-R7R8. To explore whether (1) this interaction occurs in cells and (2) whether the interaction is dependent on the presence of integrin β1, we stably expressed either full-length Caskin2 (Caskin2-FL–GFP) or a Caskin2 construct with the LD motif deleted (Caskin2-ΔLD–GFP; L1183–D1202 deleted) fused to GFP in GE11 cells expressing integrin β1 under control of doxycycline (hereafter referred to as GE11^tetON β1^ cells). GFP-Trap experiments in GE11^tetON β1^ cells showed that the LD motif is required for the interaction with talin (Fig. 1C; Fig. S1D), which occurred in both the presence and absence of doxycycline, indicating the interaction is independent of integrin β1 (Fig. S1E).

Using microscale thermophoresis (MST) we measured the affinity of the Caskin2-LD peptide to talin R7R8 as ∼2.7 µM (Fig. 1D). To further understand the mode of this interaction, we crystallized the complex and determined the crystallographic structure at 2.7 Å (1 Å=0.1 nm) resolution (Fig. 1E). The electron density map showed clear binding of the Caskin2-LD peptide to the R8 motif. The peptide was modeled, and the structure was refined to a *R*_free_ of 26.9% with excellent geometry (Table S2). The Caskin2-LD peptide was found to adopt a helical conformation that packs against two adjacent helices (α2 and α3) on the surface of the talin-R8 four-helix bundle (Fig. 1F), essentially forming a five-helix bundle. Analysis of the interaction interface (Krissinel and Henrick, 2007) showed that the buried interface area is 790 Å. The interaction is formed solely by van der Waals contacts between the talin-R8 and caskin2-LD helices, as hydrogen bonds are absent. The Caskin2 residues with the largest buried surface area are L1183 (120 Å), F1190 (113 Å), L1197 (105 Å) and L1201 (104 Å). To validate this binding mode, we transiently expressed a GFP-fused Caskin2 mutant with these four amino acids replaced by alanine (termed Caskin2-4A–GFP, Fig. S1F) in COS7 cells and performed GFP-Trap pulldown experiments. In contrast to the wild-type (WT) Caskin2–GFP construct, Caskin2-4A–GFP failed to pull down endogenous talin (Fig. 1G), confirming the interaction observed in the crystal structure.

Our structure of the binding of the Caskin2-LD peptide to talin R7R8 is very similar to the structure of the DLC1-LD peptide binding to talin R7R8 (PDB: 5FZT; Zacharchenko et al., 2016), with both forming a five-helix bundle with the R8 domain (Fig. S1G). Interestingly, the relative orientation of the R7 and R8 domains are different in the two structures, demonstrating the structural plasticity between the two talin repeats (Fig. S1H).

### Talin contains multiple Caskin2-binding sites

To investigate whether R8 is the only talin repeat recognized by the LD motif of Caskin2, we expressed GFP-tagged talin1 lacking the R8 domain (Talin-ΔR8–GFP) together with Caskin2 in COS-7 cells and performed GFP-Trap co-immunoprecipitation (co-IP) experiments. We also included in this analysis a talin mutant carrying two reverse charge mutations (R1523E and K1530E; Talin-R2E–GFP), which have previously been reported to destabilize R8 (Zacharchenko et al., 2016) and to disrupt binding of the LD-motif-containing protein DLC1 (Zacharchenko et al., 2016). Whereas Caskin2 showed only limited binding to full-length exogenous talin (owing to the presence of endogenous talin in the COS-7 cell lysates), a slightly increased binding was observed for the charge-reversal talin mutant (R1523E/K1530E), Interestingly, the strongest binding was observed for the talin ΔR8 construct (Fig. 1H). We hypothesize that the increased binding of Caskin2 to the talin ΔR8 construct might arise due to a conformational change in the talin molecule, potentially caused by disruption of the F3-R9 autoinhibition (Goult et al., 2013a) as a result of the deletion of R8, which could expose additional Caskin2-binding sites.

These findings suggest that Caskin2 is able to interact with additional talin rod domains. To provide support for this hypothesis, we used AlphaFold (Evans et al., 2022 preprint; Jumper et al., 2021) in the context of the CollabFold implementation (Mirdita et al., 2022) to model the complex between the Caskin2 LD peptide and each individual talin rod domain. This approach suggested a high-confidence complex between the peptide and the R8 domain (closely resembling our crystal structure) and a very low-confidence complex with the R7 domain, which does not bind the peptide in our structure (Fig. S2). The models also suggest that the Caskin2 LD peptide could likely form a complex with the R2, R3, R11 and R12 talin rod domains. To confirm these interactions, we used GFP-tagged talin polypeptides consisting of the R1-R3 domains (GFP–R1-R3), R5 and R6 (GFP–R5R6), R7 and R8 (GFP–R7R8), and R11-R13 including the C-terminal dimerization domain (GFP–R11-DD). GFP–Trap Co-IP experiments using these constructs together with co-expressed Caskin2 in COS-7 cells confirmed the predictions of the AlphaFold analysis, with binding observed between all of the polypeptides and Caskin2 except for GFP-R5R6 (Fig. 1I).

### Caskin2 colocalizes with members of the CMSC

**Fig. 2.**
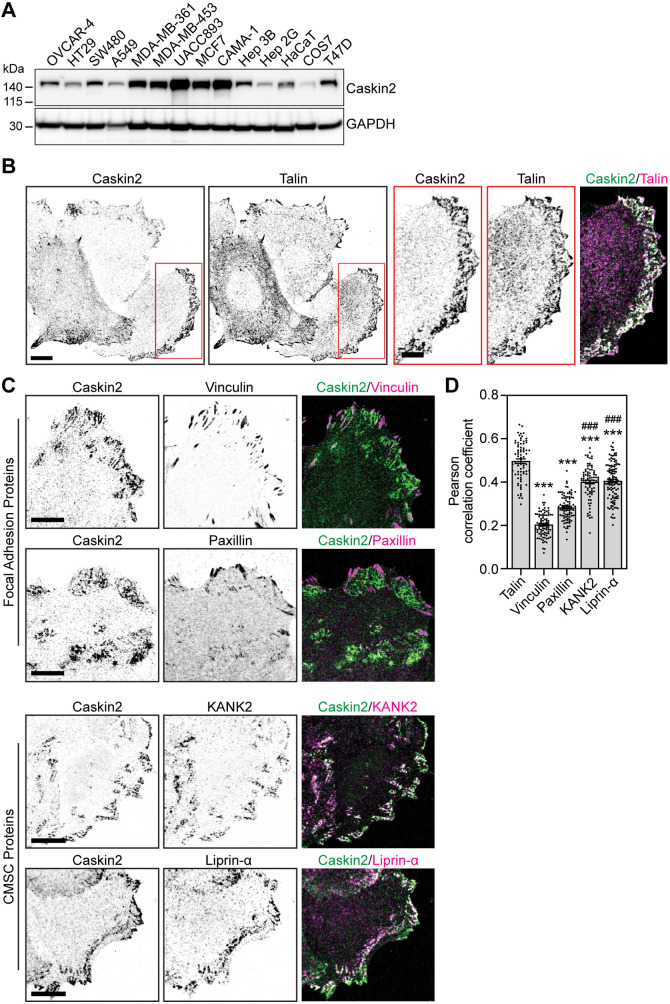
**Caskin2 localization in MCF7 breast cancer cells.** (A) Western blot analysis of whole-cell lysates from indicated cell lines, probed with antibodies against Caskin2 and GAPDH. A representative western blot is shown (*n*=3). (B) Representative immunofluorescence images showing endogenous Caskin2 (green in merge) and talin (magenta in merge), in MCF7 cells. Scale bars: 10 μm (main images); 5 µm (magnifications). Note that Caskin2 and talin do not colocalize in FAs, but colocalize in membrane-associated lattices. (C) Representative immunofluorescence images showing endogenous Caskin2 (green) together with vinculin, paxillin, KANK2 or Liprin-α (magenta) in MCF7 cells. Scale bars: 10 μm. (D) Pearson's correlation analysis for the extent of colocalization of Caskin2 with indicated FA and CMSC proteins in MCF7 cells. *n*=75 (talin); 87 (vinculin), 91 (paxillin), 83 (KANK2), and 115 (liprin-α) cells. ***P<0.0001 compared against talin; ^###^*P*<0.0001 compared against vinculin (one-way ANOVA with Holm–Šídák's multiple comparisons test).

We next examined the expression levels of endogenous Caskin2 in a variety of human cell types, including HaCaT cells, ovarian carcinoma (OVCAR-4), colorectal carcinoma (HT29 and SW480), lung adenocarcinoma (A549), breast cancer (CAMA-1, MCF7, UACC893, MDA-MB-361, MDA-MB-453 and T47D) and hepatocellular carcinoma (Hep 3B and Hep 2G). We found high levels of Caskin2 expression in MCF7 and UACC893 breast cancer cells (Fig. 2A), in agreement with previous reports that these cells contain an amplification of *CASKIN2* (Shadeo and Lam, 2006).

We examined the localization of Caskin2 in MCF7 (Fig. 2B) and UACC893 (Fig. S3A) cells using confocal microscopy. Endogenous Caskin2 localized in lattice-like structures at the cell–substratum interface, and co-staining for the FA protein talin revealed colocalization at these membrane lattices (Fig. 2B). Intriguingly, however, Caskin2 and talin did not colocalize at the ‘core’ of the FAs, with Caskin2 localized at the FA belt (Fig. 2B, Fig. S3A). In line with this finding, treatment of MCF7 cells with the myosin II inhibitor blebbistatin (10 µM) to disassemble FAs increased overall Caskin2–talin colocalization (Fig. S3B). The localization of Caskin2 to the FA belt was reminiscent of KANK1 and KANK2 localization, which are a components of CMSCs that regulate actin–microtubule crosstalk at cell–ECM adhesion sites (Astro and de Curtis, 2015; Bouchet et al., 2016; Lansbergen et al., 2006; van der Vaart et al., 2013). Examining the colocalization of Caskin2 with other markers of FAs (vinculin and paxillin, which localize to the high-tension ‘core’ of the FA) or CMSCs (KANK2 and liprin-α) revealed complete exclusion of Caskin2 from the FA core; by contrast Caskin2 colocalized with KANK2 and liprin-α, albeit to a lesser degree than with talin (Fig. 2C,D).

Tensin3, which binds directly to talin and localizes at both FAs and centrally located, tension-independent fibrillar adhesions (Atherton et al., 2022; Clark et al., 2010), was also identified as a Caskin2-proximal protein (Fig. 1B). In MCF7 cells, Caskin2 showed little to no overlap with tensin3 at peripheral FAs, whereas it co-distributed with tensin3 in centrally localized adhesions (Fig. S3C). We also observed some colocalization of integrin β5 with Caskin2 at the membrane lattices, which were distinct from integrin β5-containing flat clathrin lattices (Fig. S3D).

Based on our confocal microscopy observations, we conclude that Caskin2 is excluded from the core of the FA and is localized to the FA belt as well as adjacent to the FA together with other CMSC or plasma membrane-associated platform (PMAP) components, and at additional membrane patches. Therefore, Caskin2 is likely a novel component of the CMSC or PMAP and might function to regulate actin–microtubule crosstalk, similar to KANK1 and KANK2 (Bouchet et al., 2016; Sun et al., 2016).

### Caskin2 associates with Abi1 and other WAVE regulatory complex components

**Fig. 3.**
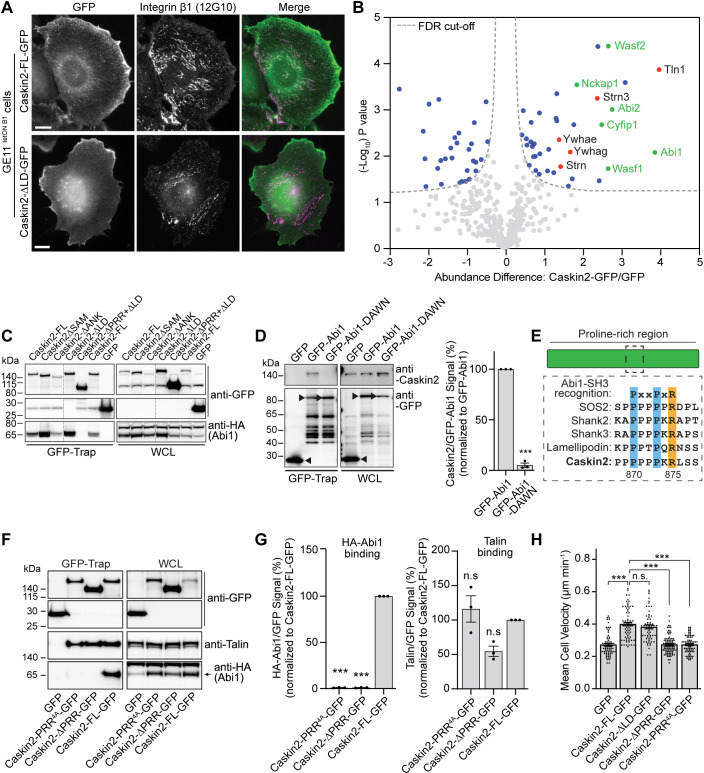
**Caskin2 interacts with components of the WRC and binds directly to Abi1.** (A) Representative immunofluorescence images from three independent repeats (∼45 cells) repeats showing Caskin2-FL–GFP and Caskin2-ΔLD–GFP expressed in GE11^tetONβ1^ cells stained for (active) integrin β1 (12G10 antibody). (B) GFP-Trap pull down of GFP (control) and GFP-tagged Caskin2 stably expressed in GE11^tetONβ1^ cells. Pulled down proteins were identified by mass spectrometry. The volcano plot shows the results from three independent experiments [false discovery rate (FDR): 0.05 and S0: 0.1]. Significant interactors of Caskin2–GFP and GFP are indicated in blue (GFP interactors on the left side and Caskin2–GFP interactors on the right side) and red, with WAVE components indicated in green. (C) GFP-Trap pulldown of COS-7 cells expressing the indicated GFP-tagged Caskin2 construct, or GFP only, together with HA–Abi1. HA–Abi1 and GFP-tagged constructs were detected in the pulldown and whole-cell lysate (WCL) samples by western blotting with antibodies against HA and GFP, respectively. (D) GFP-Trap pulldown of GFP (control) and GFP-tagged Abi1 and Abi1-DAWN transiently expressed in COS-7 cells. Caskin2 and GFP-tagged Abi1 and Abi1-DAWN in the GFP-Trap pulldown samples and WCLs were detected by western blotting with antibodies against Caskin2 and GFP. Black arrowheads indicate the expressed proteins and levels were quantified. Error bars show mean±s.d. ***P<0.001 (two-tailed unpaired *t*-test). (E) Diagram showing the PxxPxR site within the PRR of Caskin2 and homology with other known binding partners of the Abi1 SH3 domain. (F) GFP-Trap pulldown experiments using lysates of COS-7 cells co-expressing GFP (control), Caskin2-PRR^4A^–GFP, Caskin2-ΔPRR–GFP or Caskin2-FL–GFP together with HA–Abi1. Precipitated proteins were analyzed by western blotting with antibodies against GFP, talin and HA. (G) Quantification of HA–Abi1 or talin binding. Error bars are mean±s.d. ***P<0.001; n.s., not significant (one-way ANOVA with uncorrected Fisher's LSD multiple comparisons test). (H) Quantification of cell motility of GE11^tetONβ1^ cells. Error bars are mean±s.e.m.; *n*=79 (GFP), *n*=74 (Caskin2-FL–GFP), *n*=61 (Caskin2-ΔLD–GFP), *n*=89 (Caskin2-PRR^4A^–GFP), *n*=77 (Caskin2-ΔPRR–GFP) cells, pooled from two independent experiments. ***P<0.0001; n.s., not significant (Kruskal–Wallis test with Dunn's multiple comparisons test). Western blots in C, D and F are representative of three independent experiments.

To explore the function of Caskin2 in cells, we examined the localization of Caskin2-FL–GFP in the GE11^tetON β1^ cells. Caskin2-FL–GFP could be observed localizing at the cell periphery but localized only weakly at integrin β1-positive adhesion structures at the cell center (Fig. 3A). The localization at the periphery was not dependent on the talin interaction, since Caskin2-ΔLD–GFP showed a similar distribution, but lacked localization to integrin β1-positive adhesions (Fig. 3A).

This distribution was reminiscent of the talin-binding protein lamellipodin (Lee et al., 2009), which binds to Abi1 and other components of the WRC (Law et al., 2013). We explored the possibility that Caskin2 might behave similarly by using affinity purification mass spectrometry (AP-MS) to identify Caskin2-FL–GFP-binding partners. In addition to talin 1, we also identified components of the WAVE complex (including Abi1 and Abi2, Wasf1, Wasf2, Nckap1 and Cyfip1), several members of the 14-3-3 family of regulatory proteins (Ywhae, Ywhag and Ywhah), and members of the striatin-interacting phosphatase and kinase (STRIPAK) family (striatin and striatin-3) as significant hits (Fig. 3B, Table S3). Several of these interactors (ABI2, WASF1, WASF2 and NCKAP1) were also identified in AP-MS experiments conducted in HaCaT cells stably expressing Caskin2–GFP (Fig. S4A; Table S3).

Abi2, which has over 90% sequence identity with to Abi1, has been previously shown to bind to the PRR of Caskin1 (Balázs et al., 2009). To explore the possibility that Abi1 might bind directly to Caskin2, we generated Caskin2 deletion constructs (fused to GFP at their C-terminus) and co-expressed them in COS-7 cells together with HA–Abi1. Abi1 was efficiently pulled down by Caskin2-FL–GFP and deletion mutants containing the PRR; however, Caskin2 lacking the PRR did not pull down HA–Abi1 (Fig. 3C). Abi1 contains an SH3 domain that commonly recognizes proline-rich sequences (Innocenti et al., 2004; Li, 2005). A GFP-tagged Abi1 mutant harboring the mutations D453A and W455N (GFP–Abi1-DAWN), which abolishes binding of the Abi1 SH3 domain to proline-rich target proteins (Innocenti et al., 2004), was unable to precipitate Caskin2, unlike the WT GFP–Abi1 (Fig. 3D), demonstrating that the Abi1–Caskin2 interaction is mediated by the SH3 domain of Abi1.

Examination of the PRR of Caskin2 identified a type II peptide ligand similar to that found in other Abi1-binding partners including SOS1 and SOS2, Shank2 and Shank3, and lamellipodin (PxxPxR; amino acids 870–875; Fig. 3E) that could potentially be recognized by the Abi1 SH3 domain. We tested this hypothesis by using two different Caskin2–GFP constructs: Caskin2–GFP lacking amino acids 746–1035 (Caskin2-ΔPRR–GFP) and Caskin2–GFP with PxPxxPxR mutated to AxAxxAxA (Caskin2-PRR^4A^–GFP). GFP-Trap co-IP experiments in COS-7 cells showed that neither of these constructs could precipitate co-expressed HA-tagged Abi1, whereas talin binding was unaffected (Fig. 3F,G). Moreover, although Caskin2-FL–GFP colocalized with Abi1 at the cell periphery in GE11^tetONβ1^ cells, no such colocalization was observed between Abi1 and either Caskin2-PRR^4A^–GFP or Caskin2-ΔPRR–GFP (Fig. S4B). This colocalization between Caskin2-GFP and Abi1 was not affected by the loss of talin binding, since Caskin2-ΔLD-GFP showed a similar colocalization pattern as Caskin2-FL-GFP (Fig. S4B). AP-MS experiments using these same samples (Caskin2-FL–GFP versus Caskin2-PRR^4A^–GFP transiently expressed in COS-7 cells) revealed ABI1, CYFIP2 and NCKAP1 were enriched in the Caskin2-FL–GFP compared to the Caskin2-PRR^4A^–GFP sample (Fig. S4C, Table S4) suggesting the Caskin2–Abi1 interaction is responsible for an (indirect) association with other WRC components. We confirmed these findings by using GE11^tetONβ1^ cells stably expressing either Caskin2-FL–GFP, Caskin2-PRR^4A^–GFP or GFP only and performing GFP-Trap co-IP experiments. Whereas Caskin2-FL–GFP precipitated endogenous Abi1, Wasf1, Cyfip2 and Nckap1, none of these were precipitated by Caskin2-PRR^4A^–GFP; 14-3-3 proteins, several of which were also detected as potential interactors in the AP-MS experiments, precipitated with both Caskin2-FL–GFP and Caskin2-PRR^4A^–GFP (Fig. S4D).

### Caskin2 promotes cell migration via its interaction with Abi1

The WRC mediates actin polymerization downstream of Rac1 activation by activating the Arp2/3 complex, which drives the generation of branched actin filaments at cell protrusions. From our results showing that Caskin2 binds to components of the WRC, we hypothesized that Caskin2 expression might influence cell motility. We measured the migration of GE11^tetONβ1^ cells stably expressing Caskin2–GFP, Caskin2-ΔPRR–GFP, Caskin2-PRR^4A^–GFP, Caskin2-ΔLD–GFP or GFP only. Fluorescence-activated cell sorting (FACS) analysis confirmed the stable cells had similar levels of GFP and integrin β1 expression (Fig. S4E). Caskin2-FL–GFP expression increased cell motility compared to GFP-only expression by ∼45% (Fig. 3H). This affect was not mediated by the Caskin2–talin interaction, given that we observed no difference in motility between Caskin2-FL–GFP- and Caskin2-ΔLD–GFP- expressing cells (Fig. 3H). By contrast, both Caskin2-PRR^4A^–GFP- and Caskin2-ΔPRR–GFP-expressing cells migrated at a similar speed as GFP-only-expressing cells (Fig. 3H). Taken together, these experiments show that Abi1 recruits Caskin2 to the cell periphery, and that this interaction promotes cell migration.

### Phosphorylation of Caskin2 serine 878 regulates the interaction with Abi1

**Fig. 4.**
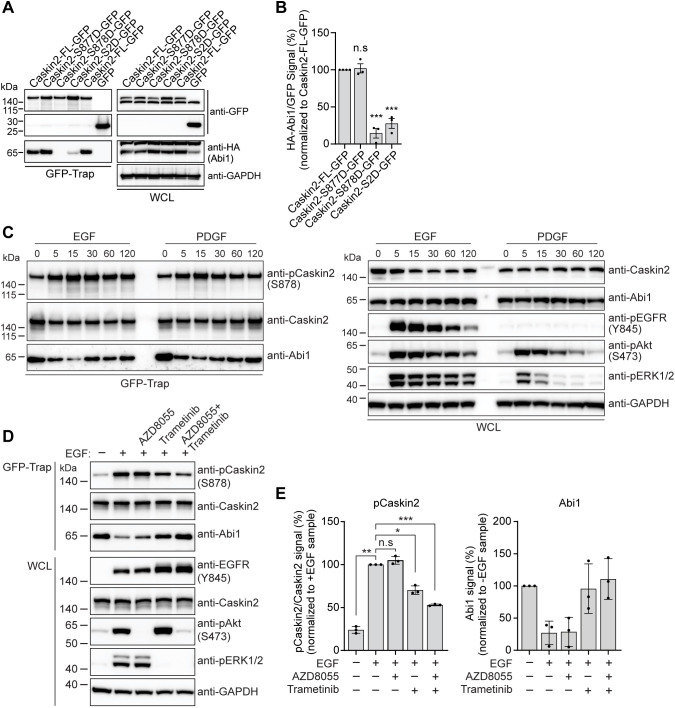
**The Caskin2–Abi1 interaction is regulated by phosphorylation of S878 of Caskin2.** (A) GFP-Trap pulldown of COS-7 cells expressing the indicated GFP-tagged Caskin2 construct or GFP only, together with HA–Abi1. HA–Abi1 and GFP-tagged constructs were detected in the pull-down and whole-cell lysate (WCL) samples by western blotting with antibodies against HA and GFP. (B) Quantification of HA–Abi1 binding. (C) GFP-Trap pull-down of GE11^tetONβ1^ cells stably expressing Caskin2-FL–GFP stimulated with either EGF (100 ng/ml) or PDGF (50 ng/ml) for the indicated time. Abi1, Caskin2 and phosphorylated (p)Caskin2 (S878) were detected in the pulldown samples by western blotting with the indicated antibodies. (D) GFP-Trap pulldown of GE11^tetON β1^ cells stably expressing Caskin2-FL–GFP stimulated with EGF (100 ng/ml for 15 min) in the presence of AZD8055 (100 nM) or Trametinib (100 nM). Abi1, Caskin2 and pCaskin2 (S878) were detected in the pulldown samples by western blotting with the indicated antibodies. (E) Graphs show quantification of pCaskin2 and Abi1 present in the GFP-Trap samples. Western blots in A, C and D are representative of three independent experiments. Error bars in B and E mean±s.d.; *P<0.05; **P<0.01; ***P<0.001; n.s., not significant (one-way ANOVA with Holm–Šídák's multiple comparisons test).

The PxxPxR SH3 domain recognition site present in Caskin2 is followed by two serine residues +2 and +3 of the arginine (S877 and S878; Fig. 3E). Intriguingly, Shank3 also has a serine at +3 relative to the arginine in the PxxPxR site, phosphorylation of which has been shown to regulate the interaction between Abi1 and Shank3 (Perfitt et al., 2020; Wang et al., 2020a). Therefore, we wondered whether serine phosphorylation plays a similar regulatory role in the Abi1–Caskin2 interaction. To test this possibility, we generated phosphomimetic mutants of Caskin2–GFP, mutating either individual serine residues only or both serine residues together to aspartic acid residues (Caskin2-S877D–GFP, Caskin2-S878D–GFP and Caskin2-S2D–GFP, respectively). Co-IP experiments in COS-7 cells expressing these constructs together with HA–Abi1 showed that HA–Abi1 binding to Caskin2-S878D–GFP was reduced by ∼80%, whereas there was no obvious difference in binding to Caskin2-S877D–GFP (Fig. 4A,B).

To explore the role of Caskin2-S878 further in regulating the Abi1 interaction, we developed a phospho-specific antibody targeting this serine. Unfortunately, high non-specific binding of the phospho-Caskin2 antibody protein sample prevented its consistent use in detecting phosphorylated Caskin2 in western blot analysis of whole-cell lysates. Only when Caskin2 was first expressed as a GFP-tagged protein and pulled down by GFP-Trap could specificity of the antibody be demonstrated. Abi1 functions downstream of growth factor receptor signaling to control Rac1-dependent actin rearrangements. Therefore, we hypothesized that Caskin2-S878 phosphorylation might occur downstream of growth factor receptor stimulation. To explore this, we conducted co-IP experiments using GE11^tetONβ1^ cells stably expressing Caskin2-FL–GFP stimulated with either EGF or PDGF. Both EGF and PDGF stimulation led to an increase in Caskin2 phosphorylation on S878 within 5 min, which was accompanied by a reduction in Caskin2–Abi1 binding (Fig. 4C). To gain insight into the kinases involved in the EGF-induced phosphorylation of Caskin2 at serine 878, we pretreated the cells with mTOR (AZD8055) and MEK1/2 (Trametinib) inhibitors for 1 h before stimulation. Both mTOR and MEK1/2 are activated downstream of EGFR activation and reduced phosphorylation of Caskin2, as detected by the phospho-specific antibody (Fig. 4D,E). However, only Trametinib, either alone or in combination with AZD8055 inhibited the EGF-stimulated loss of Abi1 binding. Taken together, our results indicate that Caskin2 can be phosphorylated at serine 878 by a serine/threonine kinase that acts downstream of the MEK1/2 kinases, which disrupts the interaction between Caskin2 and Abi1.

## DISCUSSION

We have identified Caskin2 as a novel interactor of both talin and Abi1. We show that Caskin2 interacts with the R8 domain of talin via hydrophobic residues of the LD motif at the C-terminus of Caskin2; furthermore, co-IP experiments (Fig. S1H,I) and AlphaFold modeling (Fig. S2) suggest that additional interactions can occur with the talin rod domains R2, R3, R11 and R12. Additionally, Caskin2 interacts with Abi1 via the Abi1 SH3 domain binding to the PRR of Caskin2, which connects Caskin2 indirectly to other WRC components (Fig. S4C,D). The interactions with talin and Abi1 are not mutually exclusive *in vitro*, given that disrupting the Caskin2–Abi1 interaction did not affect talin binding and vice versa (Fig. 3F,G). Mechanistically, we show that the Abi1 interaction is negatively regulated by phosphorylation of S878, which occurs downstream of MEK1/2 in response to growth factor stimulation (Fig. 4D). Intriguingly, ERK proteins have been previously shown to phosphorylate Abi1 and WAVE2, promoting the WRC–Arp2/3 interaction, thus increasing cell protrusion (Mendoza et al., 2011). Therefore, ERK-mediated phosphorylation of Caskin2 might act as an additional regulatory step in this cascade by promoting the release of Abi1 from Caskin2 facilitating WRC activation and Arp2/3 binding.

Caskin2 localization was observed in two distinct compartments: (1) at membrane ‘lattices’ in MCF7 cells, which contained talin and other CMSC components (Fig. 2); and (2) at the cell periphery when expressed as a GFP fusion protein in GE11^tetONβ1^ cells (Fig. 3). The differences in localization could arise from intrinsic differences in intracellular signaling that influence Caskin2 phosphorylation levels. Additional regulation could come from interactions of members of the 14-3-3 family of regulatory proteins and/or components of the STRIPAK signaling complex, which were consistently identified in our AP-MS experiments (Tables S1, S3, S4). Expression of Caskin2–GFP was also found to increase cell motility, a phenotype that required the interaction with Abi1, but not talin (Fig. 3H). Precisely how this occurs in response to different stimuli, and whether or how Caskin2 influences actin polymerization, will require further study.

The finding that talin colocalized with Caskin2 outside of FAs in MCF7 and UACC893 cells was surprising, particularly given the absence of other FA proteins, such as vinculin and paxillin. Current models suggest that vinculin binds talin when talin is in an active, open conformation (Goult et al., 2018), suggesting talin bound to Caskin2 at these sites might be in a closed conformation. Therefore, we speculate that Caskin2 might act to dampen the unfolding of talin rod domains in response to force. In this respect, Caskin2 would resemble KANK1 and KANK2, which are talin-interacting proteins localizing to both CMSCs and the FA belt, which act to reduce traction forces by destabilizing the integrin-actomyosin linkage (Sun et al., 2016). The talin R2, 3, 8, 11 and 12 domains contain binding sites for vinculin and actin and binding of Caskin2 to these helical bundles might act to prevent vinculin from binding, or modulate the force required to unfold the helical bundles to expose the vinculin-binding site. Force-mediated unfolding of talin rod domains might act to displace Caskin2, similar to what is observed for the talin-binding protein RIAM (Goult et al., 2013b; Vigouroux et al., 2020).

Finally, we note that both the C-terminal LD motif and the PxxPxR Abi1 interaction motif are also present in Caskin1. Caskin1 expression is restricted to neuronal tissue where it functions as a synapse protein (Katano et al., 2018), colocalizing with Shank2 and Shank3 in the post-synaptic density (PSD) (Bencsik et al., 2019). Double knockout of Caskin1 and Caskin2 disrupts the morphology of hippocampal dendritic spines, and impairs novel object recognition and spatial memory. Intriguingly, talin has been proposed to function in synaptic junctions by acting as a mechanical regulator of memory, with the unfolding of rod domains in response to force acting to control the association of scaffolding and signaling proteins (Barnett and Goult, 2022; Goult, 2021; Goult et al., 2021). Caskin1 and Caskin2 might function to control the unfolding of talin in response to force, as discussed above, and/or might link to other signaling platforms within the synapse (such as the scaffolding protein CASK for Caskin1; Smirnova et al., 2016).

## MATERIALS AND METHODS

### Construction of expression plasmids

Human Caskin2 cDNA was cloned into the *Not*I and *Sal*I sites of a pCMV-SPORT6 vector (Horizon, MHS6278_-_202759048). Caskin2 cDNA (with the stop codon removed) was assembled by *in vitro* ligation of an N-terminal part of 3.7 kb and a C-terminal PCR fragment, and cloned into the pUC19-HA-SKG269-BirA* and pUC19-HA-SKG269-GFP plasmids (Zuidema et al., 2022b), replacing the HA-tagged SKG269 DNA. Deletion mutants of Caskin2 and Caskin2-4A were generated by site-directed mutagenesis with the PCR-based overlap extension method using Pfu DNA polymerase (Promega), and fragments containing the different mutations were exchanged with corresponding fragments in the pUC19-CASKIN2-GFP plasmid. Caskin2-S877, Caskin2-S877/878D (S2D) and Caskin2-PRR^4A^ constructs were generated by purchasing gene blocks containing the desired point mutations (Genewiz) flanked with HindIII and NheI restriction sites which were used to clone the fragments into Caskin2-FL. Retroviral vectors containing mutant Caskin2–GFP and Caskin2-ΔLD cDNAs were generated by subcloning the mutant Caskin2 cDNA into the *EcoR*I and *Not*I restriction sites of LZRS-MS-IRES-Zeo (Kinsella and Nolan, 1996). Wild-type Caskin2 and Caskin2-4A were cloned into the mammalian expression vector pcDNA3 (Invitrogen) using the *EcoR*I restriction sites.

### Cell culture and generation of cell lines

Immortalized PA-JEB keratinocytes stably expressing integrin β4 (PA-JEB/β4) were generated by retroviral transduction, as previously reported (Sterk et al., 2000). Cells were maintained in keratinocyte growth medium (KGM; Invitrogen) supplemented with bovine pituitary gland extract (50 μg ml^−1^), EGF (5 ng ml^−1^), and streptomycin-penicillin (100 units ml^−1^; Sigma-Aldrich) (complete KGM). At 1 day prior to beginning the experiment, the medium was changed into Dulbecco's modified Eagle's medium (DMEM; Gibco) containing 10% fetal bovine serum (FBS; Serana Europe, Pessin, Germany) and 100 units ml^−1^ each of streptomycin and penicillin. GE11^tetONβ1^ cells were generated by infecting GE11 cells (Gimond et al., 1999) with a retroviral vector expressing integrin β1 under a tet-inducible promotor (Retro-X™ Tet-On®; Clontech, USA). Integrin β1-induced and FACS sorted cells were cultured in DMEM with 10% fetal bovine serum, containing 1 μg/ml doxycline. Human breast cancer cell line MCF7 (a gift from Lodewyk F. A. Wessels, The Netherlands Cancer Institute, The Netherlands), UACC893 (a gift from Sylvia E. Le Dévédec, Leiden University, The Netherlands) and COS-7 cells were cultured in DMEM with FCS. All cells were maintained at 37°C in a humidified atmosphere of 5% CO_2_. All cell lines were routinely tested for mycoplasma contamination every 2 to 3 months.

Stable cell lines stably expressing GFP-tagged wild-type and mutant Caskin2 constructs were generated by retroviral infection. Retroviral expression plasmids were transfected into Phoenix packaging cells using the calcium phosphate precipitation method and virus containing supernatant was collected 72 h post-transfection (Kinsella and Nolan, 1996). After infection overnight at 37°C, infected cells were selected with 0.2 mg/ml zeocin (Invitrogen) for 2 weeks (Wang et al., 2020b). GFP-positive cells were isolated by FACS (Beckman Coulter Moflo Astrios cell sorter) for following use.

For transient expression, COS-7 cells were grown to 70% confluence in 10 cm culture dishes and transfected with plasmid DNAs using the DEAE–dextran method (Schaapveld et al., 1998). Briefly, 4.5 µg of plasmid DNA was mixed with 500 µg/ml DEAE–dextran in 2.5 ml PBS. Cells were washed two times with PBS and then incubated for 30 min at 37°C with the DEAE–dextran solution. Subsequently, 80 µM chloroquine was added to the cells in 10 ml DMEM with FCS and cells were incubated at 37°C for 3 h. After aspiration of the medium, the cells were treated with 3 ml of DMEM with FCS containing 10% DMSO for 2.5 min. The DMSO solution was then removed and replaced with fresh medium, after which the cells were further incubated for 48–72 h before analysis.

### Antibodies used for immunofluorescence, western blotting and FACS

Primary antibodies used are listed in Table S5. The affinity-purified rabbit antibody selective for Caskin2 (pS787) was generated by Davids Biotechnologie (Regensburg, Germany), using the phospho-peptide PPPKRLSS(P)VSGPSPEPPPLD as immunogen. The phospho-peptide was synthesized by the protein core facility of the Max-Planck Institute (Martinsried, Germany). Rabbits were immunized with the phospho-peptide coupled to Keyhole Limpet Hemocyanon (KLH) using m-maleimidobenzoyl-N-hydroxysuccinimide (MBS). Affinity chromatography was performed using a phospho-peptide matrix. Antibodies reactive with the non-phospho version of the protein antigen were removed by passing the affinity purified antibodies over an affinity matrix with non-phospho-peptide.

Secondary antibodies for immunofluorescence were: goat anti-rabbit-IgG conjugated to Alexa Fluor 488, goat anti-mouse-IgG conjugated to Alexa Fluor 561, goat anti-rat-IgG conjugated to Texas Red and goat anti-mouse-IgG conjugated to Alexa Fluor 647 (all from Invitrogen; 1:200). Secondary antibodies for western blotting were goat anti-mouse-IgG conjugated to HRP (Bio-Rad; 1:3000) and polyclonal goat anti-rabbit-IgG conjugated to HRP (Bio-Rad; 1:5000). Secondary antibodies for FACS were goat anti-mouse-IgG conjugated to Alexa Fluor 647 (Invitrogen; 1:200).

### Immunofluorescence and image analysis

PA-JEB/β4 keratinocytes were seeded on glass coverslips and cultured for 24 h in complete KGM, and then continued to be cultured for a further 16 h in DMEM with 10% FCS. GE11^tetONβ1^ and MCF7 cells were seeded on glass coverslips and cultured for 24 h in DMEM with 10% FCS. Cells were fixed in 2% paraformaldehyde and permeabilized with 0.2% Triton X-100 for 5 min, blocked with PBS containing 2% bovine serum albumin (BSA; Sigma-Aldrich) for 45 min and subsequently incubated with primary and secondary antibodies for 1 h. In between antibody steps, the coverslips were washed three times with PBS. Actin fibers were visualized by staining with Phalloidin–Alexa Fluor 647 (Cell Signaling Technology). Nuclei were stained with DAPI. Samples were mounted on glass slides in Mowiol after three washing steps with PBS. Images were acquired with a Leica TCS SP5 confocal microscope with a 63× (NA 1.4) oil objective. Software for image acquisition is Leica LAS AF 3.0.2 (Wang et al., 2020b).

Imaging analysis was performed using FIJI software (Schindelin et al., 2012). The levels of colocalization between Caskin2 and various other proteins, including integrin β4, FA proteins and CMSC components, was performed using Pearson's co-efficient analysis in the JACoP module of ImageJ (Carisey et al., 2013; Wang et al., 2020b).

### Western blotting, immunoprecipitations and GFP-Trap assay

For analysis of protein in whole-cell lysates, cells were washed three times with cold PBS, lysed in Nonidet P-40 lysis buffer [20 mM Tris-HCl pH 7.5, 100 mM NaCl, 1% NP-40), supplemented with Na_3_VO_4_ (1.5 mM), NaF (15 mM), protease inhibitor cocktail (1:1000; Sigma-Aldrich) and phosphatase inhibitor cocktail 3 (1:100; Sigma-Aldrich)] and cleared by centrifugation at 14,000 ***g*** for 1 h at 4°C. After addition of an equal volume of 4× sample buffer (200 mM Tris-HCl pH 6.8, 10% SDS, 40% glycerol 0,1% Bromophenol Blue and 2% mercaptoethanol) and heating the samples at 95°C for 5 min, the samples were loaded on Novex NuPAGE 4–12% gradient Bis-Tris gels (Invitrogen) and transferred onto Immobilon^®^ PVDF membranes. Membranes were blocked for at least 2 h in 2% BSA in TBST (10 mM Tris-HCl pH 7.5, 150 mM NaCl with 0.05% Tween 20] before incubation with primary antibody overnight at 4°C and with secondary antibody for 1 h at room temperature. After each incubation step, the membranes were washed twice with TBST and twice with TBS (TBST without Tween 20). Finally, proteins were visualized using Clarity™ Western ECL Substrate (Bio-Rad Laboratories), as described previously (Wang et al., 2020b).

GFP-Trap pulldown experiments were performed as follows. Cleared lysates containing equal amounts of proteins, as determined by a Pierce™ BCA Protein Assay, were incubated with GFP-Trap agarose beads (Chromotek) in a rotation wheel for 6 h at 4°C. Beads were washed three times with Nonidet P-40 lysis buffer and two times with cold PBS. Bound proteins and input samples (whole-cell lysates, 1%) were then loaded on gel and transferred to Immobilon-P transfer membranes, after which the blotted proteins were probed with antibodies, as described above.

For EGF- and PDGF-BB-induced stimulation of Caskin2 phosphorylation, cells were serum starved for 16 h in DMEM without FCS. The serum-starved cells were then treated with 100 ng/ml EGF (Sigma-Aldrich) or 50 ng/ml PDGF-BB (Sigma-Aldrich) at 37°C for the indicated times. Serum-starved cells treated with EGF and the signal transduction inhibitors AZD8055 (mTOR inhibitor, Selleckchem) and Tramatinib (MEK1/2 inhibitor, Selleckchem) were first pre-treated with the inhibitor for 60 min before they were treated with the growth factor for 15 min.

### BioID assay

PA-JEB/β4 cells stably expressing Caskin2-BirA* fusion proteins were grown to 70–80% confluence on 3×145 mm plates in biotin-depleted KGM before they were treated with 50 μM biotin (Sigma-Aldrich #B4501) for 20 h at 37°C in DMEM with 10% FCS. Biotinylated proteins were captured with streptavidin–Sepharose beads (GE Healthcare) and bound proteins analyzed by mass spectrometry.

### MST measurements

MST measurements were carried out using premium-coated capillaries to reduce non-specific interaction of the proteins with the glass surface on a Monolith NT.115 red-blue (Nanotemper, Munich, Germany), as described previously (Zuidema et al., 2022b). Peptides and proteins were transferred into MST buffer (20 mM Tris-HCl pH 7.5, 200 mM sodium chloride, 1 mM TCEP and 0.05% Tween-20) to avoid artifacts derived from buffer mismatches. Atto488-labeled Caskin2-LD and scrambled peptide (synthesized by the MPIB Core Facility) were used as ligands at a concentration of 50–200 nM and added in a 1:1 ratio to a 1:1 serial dilution of recombinant R7R8 talin protein. The measurements were performed at 10 to 20% LED power and 20 and 40% MST power, and finally analyzed using the MO Affinity Analysis Software (Nanotemper).

### Recombinant protein expression and purification

A codon-optimized synthetic gene encoding murine talin-R7/R8 domains (residues 1358–1653) was cloned into the pETNKI-6xhis-3C-LIC vector (Luna-Vargas et al., 2011). The 6×his–3C–talin-R7/R8 protein was expressed in Bl21(DE3) cells, for 18 h at 20°C upon induction with 0.4 mM IPTG. Cells were harvested by centrifugation (4000 ***g*** for 15 min) and stored at −20°C. After thawing, cells were resuspended in lysis buffer [25 mM Tris-HCl pH 8.0, 10 mM imidazole, 200 mM NaCl, 1 mM TCEP and 5 μg/ml DNase (Roche)] and lysed by sonication. The soluble lysate fractions were collected after centrifugation (55,000 ***g*** at 4°C for 30 min) and applied to a nickel Sepharose column. Beads were washed with wash buffer (lysis buffer without DNase) and protein was eluted in the same buffer supplemented with 250 mM imidazole. The 6×His tag was cleaved off by his-3C protease during dialysis against 25 mM Tris-HCl pH 8.0, 100 mM NaCl, 1 mM TCEP for 16 h at 4°C. The protein solution was diluted with an equal volume of 25 mM Tris-HCl pH 8.0, 1 mM TCEP before his-3C protease and uncleaved 6×his–talin-R7/R8 were removed by nickel Sepharose. The protein was further purified by anion exchange chromatography (Resource Q column, Cytiva) followed by size exclusion chromatography (S75 Superdex 16/60, Cytiva) in 25 mM Tris-HCl pH 8.0, 100 mM NaCl, 1 mM TCEP. Talin-R7/R8 eluted in a single peak. The protein was concentrated to 32 mg/ml and aliquots were snap-frozen in liquid nitrogen and stored at −80°C.

### Talin-R7/R8–Caskin2-LD peptide crystallization

Purified talin-R7/R8 protein (32 mg ml^−1^) was mixed with solubilized Caskin2 LD peptide in a 1:2 molar ratio of protein to peptide. Crystallization screening was performed using the vapor diffusion sitting-drop method; droplets of 200 nl, composed of equal volumes of protein complex and crystallization agent, were prepared using a Mosquito dropsetter (SPT Labtech) as described previously (Newman et al., 2005). Conditions revealing initial crystal growth were further optimized using a Formulator liquid handler (Formulatrix). Diffracting crystals were grown in 0.1 M bis-Tris propane pH 7.0, 0.1 M NaBr and 22–26% PEG3350. Crystals were cryo-protected by increasing the PEG3350 concentration to 30% and by addition of 10% glycerol before vitrifying them in liquid nitrogen.

### X-ray crystallography structure analysis

Crystallographic data were collected at the PX1 beamline at the Swiss Light Source (SLS), Villigen, Switzerland and processed with XDS (Kabsch, 2010) and Aimless (Evans and Murshudov, 2013). The phase problem was solved by molecular replacement using Phaser (McCoy et al., 2007) and two separate search models, using the Talin R7 and R8 domains (PDB: 5FZT) (Zacharchenko et al., 2016). After a few cycles of model building in Coot (Emsley et al., 2010) and model refinement in REFMAC5 (Kovalevskiy et al., 2018), the electron density for Caskin-2 peptide was clearly visible. Owing to the lower local resolution of the map at that area, five probable alternative registers were modeled and refined. Additionally, the register was sought independently with the ‘Assign sequence’ function in Coot. Both approaches led to the same register. The structure model was finalized by performing alternating cycles of model building in Coot, validation in MolProbity (Chen et al., 2010) and Tortoize (van Beusekom et al., 2018), and refinement in PDB-REDO using homology restraints (van Beusekom et al., 2018). Data collection and model statistics are reported in Table S2.

### Modeling of the interaction of the Caskin2 LD peptide in complex with talin rod domains

The ColabFold interface to AlphaFold (Mirdita et al., 2022) and AlphaFold Multimer (Evans et al., 2022 preprint; Jumper et al., 2021) were used to model the interactions of the Caskin2 LD peptide with all 13 complexes with the talin rod domains. The sequence of the peptide was used as ‘chain A’ and that of each rod domain as ‘chain B’. An artificial sequence for R7 was constructed by fusing the two sequence fragments that make up those helical fragments, based on our crystal structure. The confidence of the interactions was judged by manual inspection of the Predicted Alignment Error (PAE) plots, which is a useful metric to assess how confident the model is about the interface. The modeling procedure could reproduce our crystal structure of the R8 domain with very high confidence in all five top-ranked models, whereas it only produced very low confidence interfaces with the R7 domain (which does not bind the LD peptide in our structure), indicating that this is a suitable protocol to find additional interactors. High confidence complex interfaces were predicted for all five top-ranked models for the complexes with R3, R11 and R12, while two high-confidence models were available for the R2 complex.

### Mass spectrometry

As described in our previous work (Wang et al., 2020b), samples from BioID, GFP-Trap and peptide pull-down assay were separated on a 4–12% SDS–PAGE gel. The gel was stained with Coomassie Blue and lanes were excised and then reduced by treating with dithiothreitol and alkylated with iodoacetamide. After digestion with trypsin (mass spec grade, Promega), peptides were extracted with acetonitrile. A vacuum centrifuge was used to dry the digests, which were re-suspended in 10% formic acid. Peptides were analyzed by nanoLC-MS/MS on an Orbitrap Fusion Tribrid mass spectrometer equipped with a Proxeon nLC1000 system (Thermo Fisher Scientific). Subsequently, samples were eluted from the analytical column at a constant flow of 250 nl min^−1^ in a 140-min gradient, containing a 124-min linear increase from 6% to 30% solvent A (0.1% formic acid in water), followed by a 16 min wash at 100% solvent B (0.1% formic acid and 80% acetonitrile).

For data analysis, we used either the human Swissprot database (20,432 entries, release 2019_09) or the *Mus musculus* Swissprot database (17,141 entries, release 2023_03), as data sources and applied MaxQuant (version 2.4.2.0; Cox et al., 2014) with standard settings to our raw data for label-free quantitation (LFQ). MS/MS data were concatenated with the reversed version of all sequences from the database. Trypsin/P was chosen as cleavage specificity allowing two missed cleavages; carbamidomethylation (C) was set as a fixed modification, whereas oxidation (M) was used as variable modification. LFQ intensities were Log2-transformed in Perseus (version 1.6.7.0; Tyanova et al., 2016), after which proteins were filtered for at least two valid values (out of three total). Missing values were replaced by imputation based a normal distribution using a width of 0.3 and a downshift of 1.8. Differentially expressed proteins were determined using a two-tailed unpaired *t*-test.

### Cell migration

Cell motility assays were conducted using time-lapse recordings of cells cultured on plastic 12-well plates in DMEM containing 1% FCS and 1% antibiotics. Phase-contrast images were acquired using a Zeiss AxioObserver Z.1 microscope equipped with a heated stage (37°C and 5% CO_2_), with a 10×/0.30 EC Plan-Neofluar Ph1 objective (Zeiss), captured with a Zeiss AxioCam MRm. Images were acquired every 10 min for 20 h. Cells were tracked manually using ImageJ; tracking data was analyzed using the Chemotaxis Tool plugin (Ibidi).

### Statistical analysis

The two-sided unpaired Student's *t*-test was used to calculate significance between two groups using GraphPad Prism 10 (La Jolla). Data distribution was tested using the D'Agostino-Pearson normality test with a significance levels of 0.05. Graphs were made in GraphPad Prism; statistically significant values are indicated as *P<0.05; **P<0.01; ***P<0.001; ****P<0.0001.

## Supplementary Material

10.1242/joces.262116_sup1 Supplementary information

Table S3. GE11 and HaCaT GFP-Trap mass spectrometry.

Table S4. COS-7 GFP-Trap mass spectrometry.
