# Supplementary material for: Caskin2 is a novel talin- and Abi1-binding protein that promotes cell motility
Source: J Cell Sci. 2024 May 16;137(9):jcs262116. doi: 10.1242/jcs.262116 (PMC11166458; doi:10.1242/jcs.262116)
Supplement: Supplementary information [file joces-137-262116-s1.pdf]

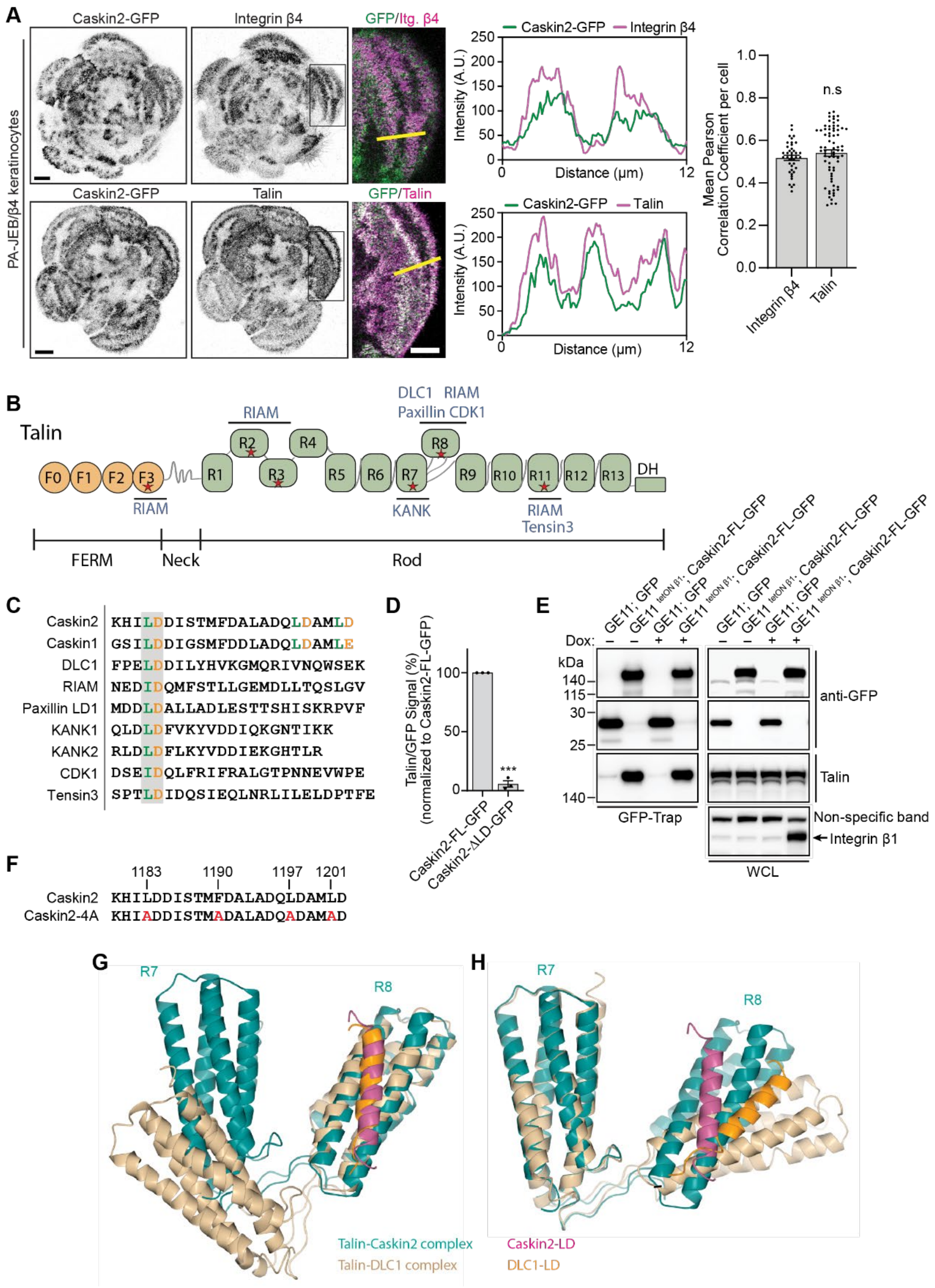

Fig. S1. Caskin2 co-distributes with talin and localizes in the proximity of HDs, FAs and CMSCs. (***A***) Representative immunofluorescence images show Caskin2-GFP (green) together with integrin β4 or talin (magenta) in PA-JEB/β4 keratinocytes. Scale bar, 10 μm; in inserts represents 5 μm. Line profiles shows Caskin2 co-localises with both talin and integrin β4. Graph

Journal of Cell Science • Supplementary information

shows the mean Pearson Correlation Coefficient per cell between the Caskin2-GFP signal and either Integrin β4 or talin; n.s indicates no significant difference; error bars are standard error; unpaired t-test; n = 40 (Integrin β4) and 71 cells (talin) pooled from 3 independent experiments. (***B***) Talin comprises of a FERM domain with four subdomains (F0-F3, in orange) and a rod domain with 13 bundles (R1-R13, in green), followed by a C-terminal dimerization helix (DH). Some critical interacting proteins are indicated. (***C***) Sequence alignment of the talin-binding motifs of Caskin2, Caskin2, DLC1, RIAM, Paxillin LD1, KANK1, KANK2 and CDK1. Acidic (orange) and hydrophobic (green) residues that mediate the interaction are highlighted. (***D***) Quantification of Talin binding to the indicated GFP-tagged construct, normalized to Caskin2-FL-GFP (corresponding to the graph in Figure 1C). Values were quantified from 3 independent Western blots, error bars are standard error, *** indicates $p<0.0001$ (unpaired t-test). (***E***) GFP-Trap pull-down of GFP expressed in wild-type GE11 (β1-null) or Caskin2-FL-GFP expressed in GE11 $^{tetON\ \beta1}$ cells. Cells were cultured continually with or without doxycycline as indicated. Proteins in the GFP-Trap sample and WCL (input) are detected by Western blotting, using antibodies against GFP, talin, and integrin β1. Note that doxycycline induces integrin β1 expression only in the GE11 $^{tetON\ \beta1}$ cells. Talin binding to Caskin2-FL-GFP is not affected by the presence of integrin β1. A representative Western blot is shown (n=3). (***F***) Amino acid sequences of the Caskin2 LD-motif and the 4A mutant containing 4 alanine mutations. (***G** and **H***) Comparison of the crystal structure of talin R7R8 in complex with Caskin2-LD (magenta) and in complex with DLC1-LD (residues 467-489; orange), which is aligned on the R8 domain (***G***) and R7 domain (***H***).

Journal of Cell Science • Supplementary information

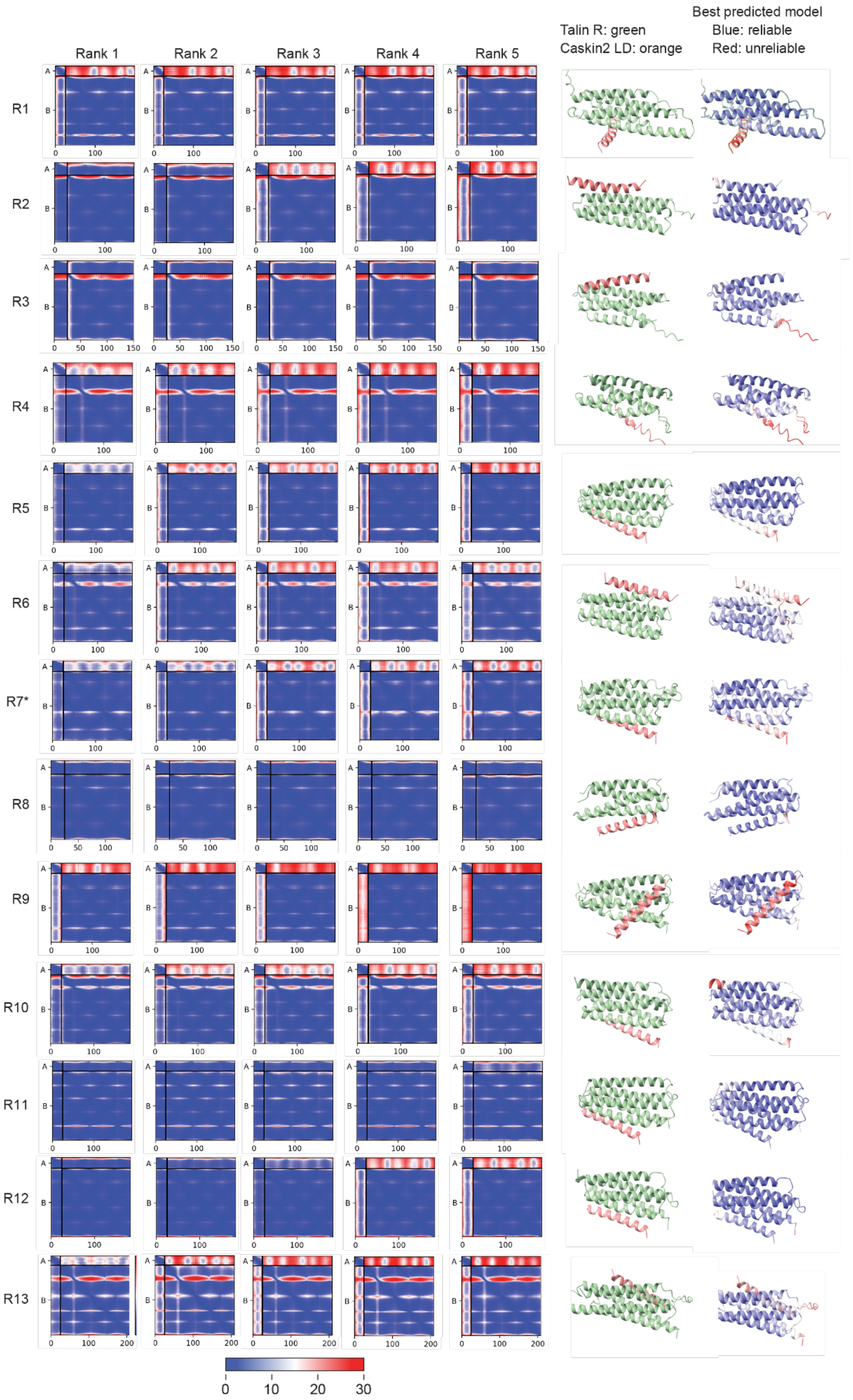

Fig. S2. AlphaFold modelling for Caskin2-LD interactions with different talin rod domains. The different columns show: the talin rod domain examined in each row; the Predicted Alignment Error (PAE) plots for the five top-ranked models (blue: high confidence interaction; red: low confidence interaction); a cartoon model of the complex with talin rod domain in green and the Caskin2 LD peptide in orange; the same model coloured by local confidence.

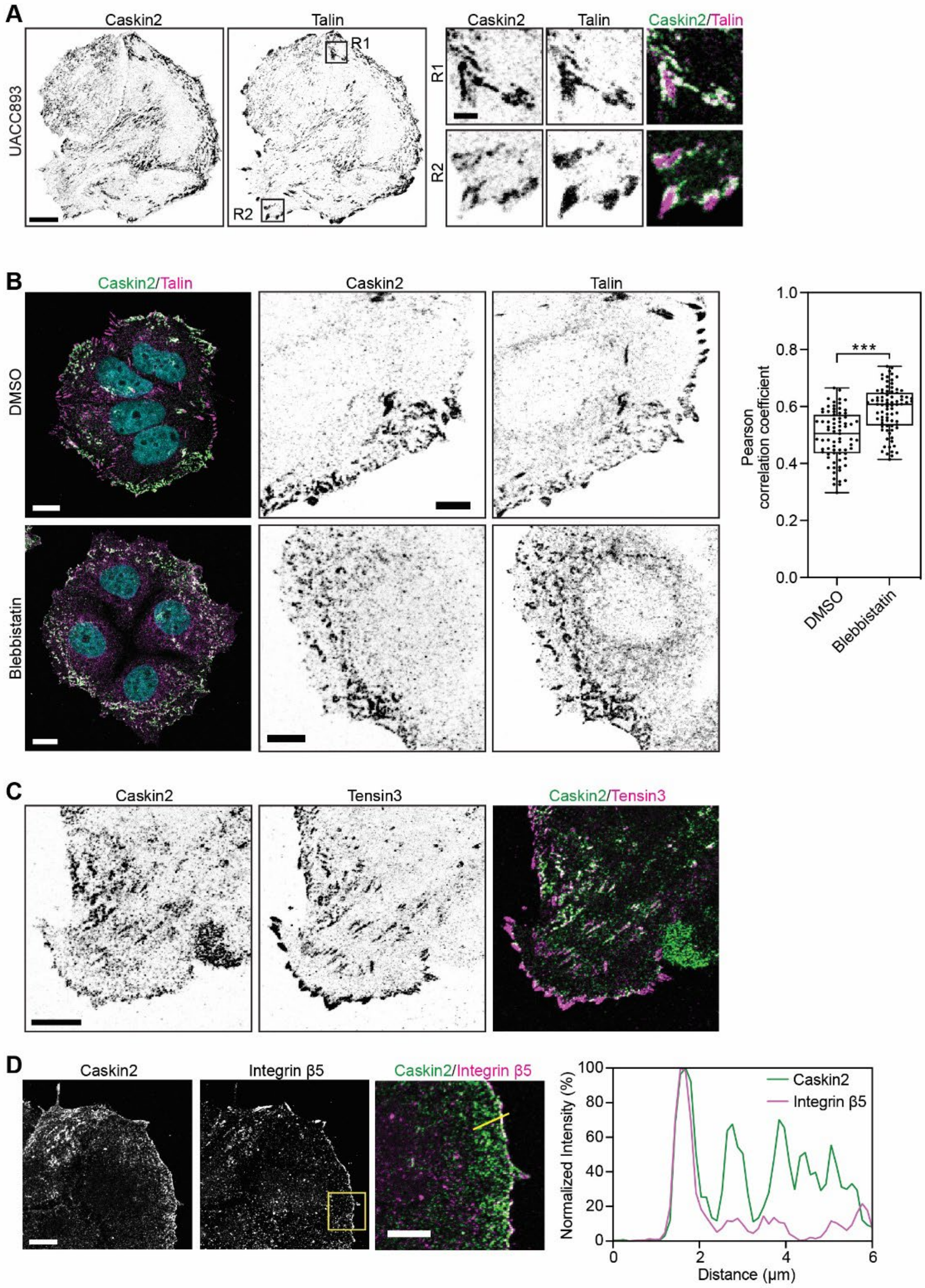

Fig. S3. Representative IF images show endogenous Caskin2 (green in merge) and talin (magenta in merge), in UACC893 cells. Scale bar, 10 μm; in inserts represents 2 μm. (***B***) Representative IF images show Caskin2 (green), talin (magenta), and DAPI (cyan) in MCF7 cells treated with DMSO or 20 μM blebbistatin for 45 min. Scale bar, 10 μm; in magnified region, 5 μm. Pearson's correlation analysis for the extent of co-localization of Caskin2 with talin in DMSO (n = 75) or Blebbistatin (n = 81) treated MCF7 cells. Data are presented as box-and-whisker plots, in which the box extends the 25th to 75th

Journal of Cell Science • Supplementary information

percentiles, the middle line indicates the median, and whiskers go down to the smallest value and up to the largest. ****, $P < 0.0001$, t-test. (***C***) Representative IF images showing endogenous Caskin2 (green) together with Tensin3 (magenta) in MCF7 cells. Scale bar, 10 μm. (***D***) Representative IF images showing endogenous Caskin2 (green in merge) and integrin β5 (magenta in merge) in MCF7 cells. Yellow arrow shows integrin β5 in flat clathrin lattices, which do not contain Caskin2. Scale bar indicates 10 μm.

Journal of Cell Science • Supplementary information

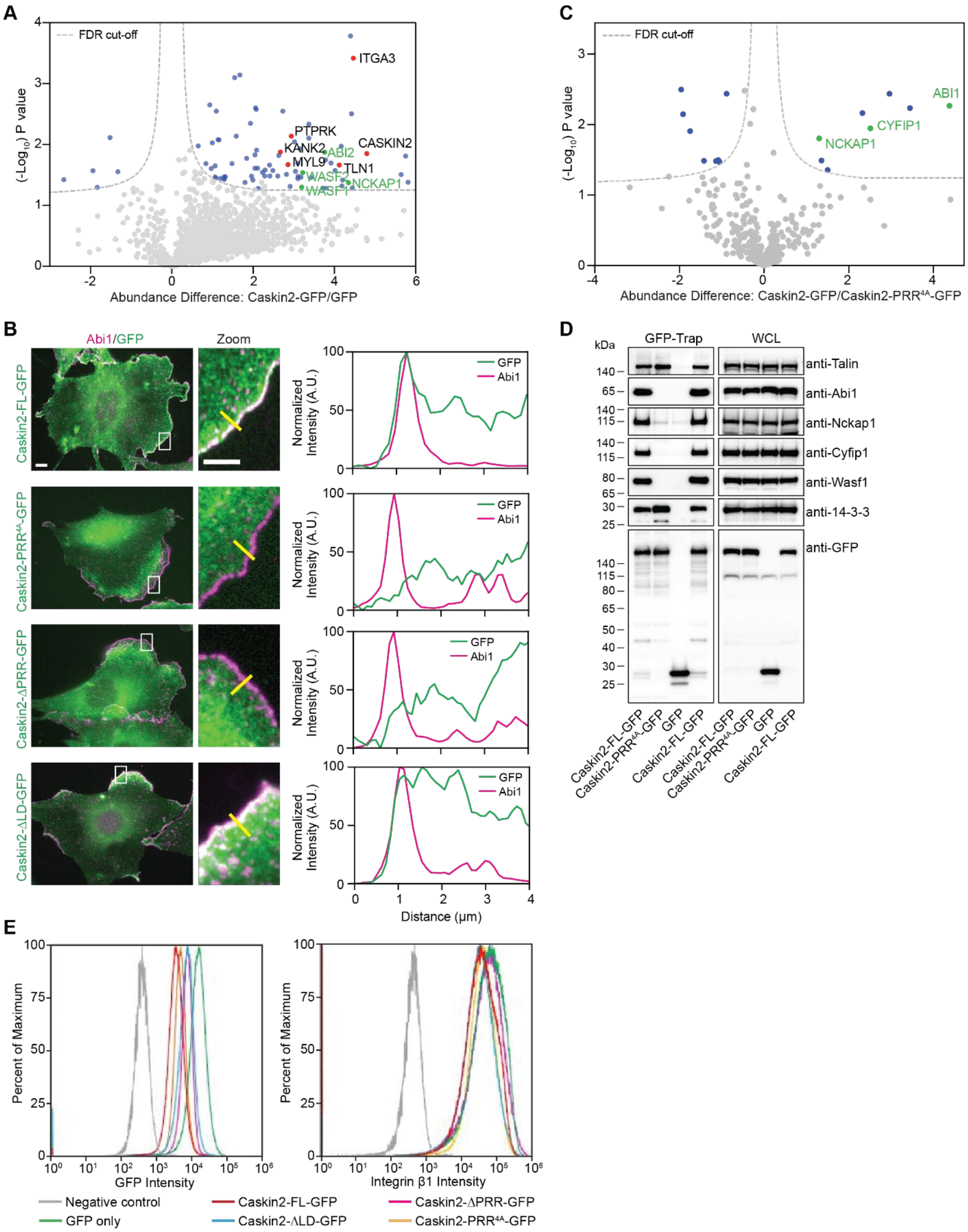

Fig. S4. Caskin2 associates with components of the WAVE Regulatory Complex. (*A*) GFP-Trap pull-down of GFP (control) and GFP-tagged Caskin2 stably expressed in HaCaT cells. Pulled down proteins were identified by mass spectrometry. The volcano plot shows the results from three independent experiments (FDR: 0.05 and S0: 0.1). Significant interactors of Caskin2 and GFP

Journal of Cell Science • Supplementary information

are indicated in blue (GFP interactors on the left side and Caskin2 interactors on the right side) and red, with WAVE components indicated in green. (***B***) Representative immunofluorescence images of GE11 $^{tetON\ \beta1}$ cells expressing Caskin2-FL-GFP, Caskin2-PRR^{4A}-GFP, Caskin2-ΔPRR-GFP, or Caskin2-ΔLD-GFP together with endogenous Abi1. Line profiles show Caskin2-FL-GFP co-localises with Abi1 at the cell periphery, which is not seen in the two constructs deficient for Abi1 binding. Deletion of the talin-binding LD motif does not affect Abi1 co-localization. Scale bars indicate 10 µm; in magnified regions indicate 5 µm. (***C***) GFP-Trap pull-down of Caskin2-FL-GFP and Caskin2-PRR^{4A}-GFP co-expressed with HA-Abi1 in COS-7 cells. Pulled down proteins were identified by mass spectrometry. The volcano plot shows the results from three independent experiments (FDR: 0.05 and S0: 0.1). Significant interactors of Caskin2-FL-GFP but not Caskin2-PRR4A-GFP are on the right side indicated in blue; WAVE components are indicated in green. (***D***) GFP-Trap pull-down of GFP (control), Caskin2-FL-GFP or Caskin2-PRR^{4A}-GFP stably expressed in GE11 $^{tetON\ \beta1}$ cells. Precipitated proteins were analyzed by Western blotting, probed with antibodies against GFP, Talin, Abi1, Nckap1, Cyfip2, Wasf1 and 14-3-3. (***E***) Flow cytometry analysis of GFP and integrin β1 expression in GE11 $^{tetON\ \beta1}$ cells stably expressing the indicated construct.

Table S1. Caskin2 BioID in PA-JEB/β4 keratinocytes.

Available for download at
https://journals.biologists.com/jcs/article-lookup/doi/10.1242/jcs.262116#supplementary-data

Table S2. Data collection and refinement statistics for protein structure analysis.

**Data Collection**	
Wavelength (Å)	1.00
Resolution (Å)	49.09−2.72 (2.85−2.72)[a]
Space Group	$P\ 2\ 2_1\ 2_1$
Unit Cell a, b , c (Å)	46.66, 61.35, 98.18
$CC_{1/2}$	0.998 (0.605)[a]
R_{merge}	0.162 (2.479)[a]
<I/σ(I)>	9.5 (1.3)[a]
Completeness (%)	99.8 (99.0)[a]
Multiplicity	12.9 (13.6)[a]
**Refinement**	
Reflections work/test (nr)	7581/424
Atoms Talin/Caskin-2 (nr)	2189/193
B-factors Talin/Caskin-2 (Å^2)	87/77
$R_{work}/R_{free}/R_{complete}$[b] (%)	24.2/26.9/27.9
rmsZ bond lengths/bond angles[b]	0.349/0.530
Model validation	
Ramachandran preferred/outliers[c] (%)	99.1/0.0
Ramachandran Z-score[d]	-1.37 ± 0.50
Rotamers preferred/outliers (%)[c]	99.2/0.4
Rotamer Z-score[d]	-1.59 ± 0.57
Clashscore (%-ile)[c]	100
MolProbity score (%-ile)[c]	100

Table S3. GE11 and HaCaT GFP-Trap mass spectrometry.

Available for download at
https://journals.biologists.com/jcs/article-lookup/doi/10.1242/jcs.262116#supplementary-data

Table S4. COS-7 GFP-Trap mass spectrometry.

Available for download at
https://journals.biologists.com/jcs/article-lookup/doi/10.1242/jcs.262116#supplementary-data

Table S5. Primary antibody list

Antibody	Clone/Cat#	Obtained from	Host	Application
Integrin β1	12G10	Prof. M. Humphries[1]	Mouse mAb	FACS: 1:200
Integrin β4	450-11A/555722	BD Bioscience	Mouse mAb	IF: 1:100
Integrin β5	5HK2	Homemade[2]	Rabbit pAb	WB: 1:1000
Caskin2	HPA075184	Sigma-Aldrich	Rabbit pAb	IF: 1:100<br>WB: 1:1000
Caskin2	D9/sc-377512	Santa Cruz Biotechnology	Mouse mAb	IF: 1:100<br>WB: 1:1000
Talin	8D4/157808	Abcam	Mouse mAb	IF:1:200<br>WB: 1:1000
Vinculin	VIIF9 (supernatant)	Dr. M. Glukhova[3]	Mouse mAb	IF: 1:5
Paxillin	Clone 349/P13520	BD transduction Lab.	Mouse mAb	IF: 1:100
Tensin3	PA5-63112	ThermoFischer Scientific	Rabbit pAb	IF: 1:100
KANK2	HPA015643	Sigma-Aldrich	Rabbit pAb	IF: 1:100
Liprin α1	14175-1-AP	Proteintech	Rabbit pAb	IF: 1:200
Abi1	HPA068407	Sigma, Atlas antibodies	Rabbit pAb	WB: 1:1000<br>IF: 1:100
WAVE	F10/sc-365165	Santa Cruz Biotechnolgy	Rabbit pAb	WB: 1:1000
NCKAP1	PA5-88433	InVitrogen	Rabbit pAb	WB: 1:1000
CYFIP1	PA5-87780	InVitrogen	Rabbit pAb	WB: 1:1000
14-3-3		Homemade[4]	Rabbit pAb	WB: 1:1000
pEGFR (Y845)	#2231	Cell Signaling Technology	Rabbit pAb	WB: 1:1000
pAkt (S473)	#9271S	Cell Signaling Technology	Rabbit pAb	WB: 1:1000
pERK1/2 (p44/p42) (T202/Y204)	#9101	Cell Signaling Technology	Rabbit pAb	WB: 1:1000
GAPDH	D16H11/#5174	Cell Signaling Technology	Rabbit mAb	WB: 1:1000
GFP		Homemade[5]	Rabbit pAb	WB: 1:1000
HA	12CA5	ThermoFischer Scientific	Mouse mAb	WB: 1:1000

1. Prof. M. Humphries, University of Manchester, Manchester, UK
2. Zuidema, A., Wang, W., Kreft, M., te Molder, L., Hoekman, L., Bleijerveld, O.B., Nahidiazar, L., Janssen, H., and Sonnenberg, A. (2018). Mechanisms of integrin αVβ5 clustering in flat clathrin lattices. J. Cell Sci. 131(21):jcs221317. doi: 10.1242/jcs.221317.
3. Dr. M. Glukhova, Institute Curie, Paris, France
4. Van der Hoeven, P.C.J., van der Wal, J.C.M., Ruurs, P., van Dijk, M.C.M., and van Blitterswijk, W.J. (2000). 14-3-3 Isotypes facilitate coupling of protein kinase C-ζ to Raf-1 : negative regulation by 14-3-3 phosphorylation. Biochem. J. *345*, 297–306.
5. Rocha, N., Kuijl, C., van der Kaant, R., Janssen, L., Houben, D., Janssen, H., Zwart, W., and Neefjes, J. (2009). Cholesterol sensor ORP1L contacts the ER protein VAP to control Rab7–RILP–$p150^{Glued}$ and late endosome positioning. J. Cell Biol. *185*, 1209-1225.

Journal of Cell Science • Supplementary information
